# Provider perspectives on PrEP for adolescent girls and young women in Tanzania: The role of provider biases and quality of care

**DOI:** 10.1371/journal.pone.0196280

**Published:** 2018-04-27

**Authors:** Nanlesta Pilgrim, Nrupa Jani, Sanyukta Mathur, Catherine Kahabuka, Vaibhav Saria, Neema Makyao, Lou Apicella, Julie Pulerwitz

**Affiliations:** 1 Population Council, Washington, DC, United States of America; 2 CSK Research Solutions, Dar es Salaam, Tanzania; 3 National AIDS Control Programme, Dar es Salaam, Tanzania; UNAIDS, UNITED STATES

## Abstract

**Background:**

Oral pre-exposure prophylaxis (PrEP) has the potential to reduce HIV acquisition among adolescent girls and young women (AGYW) in sub-Saharan Africa. However, health care providers’ (HCPs) perspectives and interactions with potential clients can substantially influence effective provision of quality health services. We examine if HCPs’ knowledge, attitude, and skills, as well as their perceptions of facility readiness to provide PrEP are associated with their willingness to provide PrEP to AGYW at high risk of HIV in Tanzania.

**Methods:**

A self-administered questionnaire was given to 316 HCPs from 74 clinics in two districts and 24 HCPs participated in follow-up in-depth interviews (IDIs). We conducted bivariate and multivariable Poisson regression to assess factors associated with willingness to provide PrEP to AGYW. Thematic content analysis was used to analyze the IDIs, which expanded upon the quantitative results.

**Results:**

Few HCPs (3.5%) had prior PrEP knowledge, but once informed, 61.1% were willing to prescribe PrEP to AGYW. Higher negative attitudes toward adolescent sexuality and greater concerns about behavioral disinhibition due to PrEP use were associated with lower willingness to prescribe PrEP. Qualitatively, HCPs acknowledged that biases, rooted in cultural norms, often result in stigmatizing and discriminatory care toward AGYW, a potential barrier for PrEP provision. However, better training to provide HIV services was associated with greater willingness to prescribe PrEP.

Conversely, HCPs feared the potential negative impact of PrEP on the provision of existing HIV services (e.g., overburdened staff), and suggested the integration of PrEP into non-HIV services and the use of paramedical professionals to facilitate PrEP provision.

**Conclusions:**

Preparing for PrEP introduction requires more than solely training HCPs on the clinical aspects of providing PrEP. It requires a two-pronged strategy: addressing HCPs’ biases regarding sexual health services to AGYW; and preparing the health system infrastructure for the introduction of PrEP.

## Introduction

Oral pre-exposure prophylaxis (PrEP), the use of antiretroviral medications by HIV-uninfected persons to prevent HIV, has the potential to substantially reduce HIV acquisition among adolescent girls and young women (AGYW) aged 15–24, a population that accounts for 25% of new HIV infections among adults in sub-Saharan Africa [1]. In Tanzania, the prevalence of HIV is 1.3% and 4.4% among adolescent girls aged 15–19 and young women aged 20–24, respectively [2]. PrEP has been shown in over 11 randomized control trials to substantially reduce risk of HIV infection with very high adherence [3, 4]. In 2015, the World Health Organization (WHO) released guidelines noting that PrEP should be offered to individuals at substantial risk for HIV as part of combination prevention approaches [3, 4]. Yet, PrEP efficacy among AGYW will be dependent on whether they can effectively access, use, and adhere to it.

Access, use, and adherence to PrEP requires substantial commitment and support from health care providers (HCPs), whose perspectives and experiences occur within the context of broader structural and facility factors that may influence their decision-making about who and how to provide PrEP [5–10]. PrEP requires a prescription, specialized counseling, regular HIV testing, and close clinical monitoring for side effects and seroconversion through frequent follow-up [11, 12]. HCPs’ commitment, close clinical monitoring, and support become even more essential for AGYW as research shows that women and younger participants in PrEP trials are less likely to adhere to the medication [13–16]. Moreover, adolescents and young adults are often reluctant to seek sexual and reproductive health services due to experiences of poor quality of care and stigma and discrimination from HCPs [17–19]. Thus, it is important to gauge HCPs’ perceptions and concerns about providing PrEP to AGYW and their views on facility-level factors that may influence how they provide PrEP services to AGYW. However, little evidence exists on the knowledge, attitudes, and perception of HCPs’ toward PrEP in sub-Saharan Africa and nothing is known about HCPs’ perceptions toward PrEP for AGYW in Tanzania [12, 20]. Furthermore, because PrEP is still a relatively new biomedical intervention, it is necessary to understand the issues surrounding its implementation into health care systems and HCPs are key to this implementation.

In this study, we utilized mixed methods to assess factors influencing HCPs’ willingness to provide PrEP to AGYW at high risk of HIV in Tanzania. Drawing upon prior work [5–8, 10, 21, 22], including the WHO global standards for quality healthcare services for adolescents [18], we apply a quality of care framework to distill key aspects of provider- and facility-level factors that influences HCPs’ perceptions, readiness, and willingness to provide PrEP to AGYW. We distilled the framework, which consists of eight domains, into provider and facility levels [5–10]. At the provider-level, the framework captures (1) patient-centered care—care is tailored to the needs and preferences of individual clients, including the provision of equitable, non-stigmatizing, and non-discriminatory care; (2) client-staff interactions—providers and staff demonstrate respect, courtesy, friendliness and empathy, and respect clients’ privacy; (3) communication and information—information provided to clients is understandable and sufficient; and (4) technically competent care—care provided is safe, effective, and complies with accepted clinical standards. At the facility-level, the framework captures (5) accessibility—care is geographically accessible, affordable, and convenient; (6) efficient and effectively organized care—care in organized in terms of waiting time, follow-up, and referrals; (7) structure and facilities—comfortableness, safety, cleanliness, and privacy of facilities; and (8) appropriate package of services—package of services (e.g., information, counseling) is provided that fulfill the needs of adolescents and young adults.

## Methods

### Study design

The study takes place in a rural district in the Mbeya region, and in an urban district in the Dar es Salaam region. We utilized a mixed-methods approach, which allows for cross-validation and triangulation of findings. Data collection occurred between March and June 2017 for both the qualitative and quantitative components of the study.

### Quantitative component

#### Sampling

We obtained a complete list of all public and private health facilities offering HIV prevention, care, treatment, and support services from each district’s health office. We identified a total of 69 facilities in Dar es Salaam and 36 in Mbeya, which were categorized as hospitals, health centers/clinics, and dispensaries. Except for dispensaries in the Dar es Salaam region, all facilities in Dar es Salaam and Mbeya were included in the survey. Dar es Salaam is a larger district and had considerably more dispensaries than Mbeya. Given available resources, we randomly selected half of the dispensaries in Dar es Salaam for inclusion to ensure representativeness. A total of 74 facilities across the two sites were included in the final sample; 9% were hospitals, 15% were health centers, and 76% were dispensaries. Eligible HCPs present at the facility on the day of data collection completed the survey. HCPs were eligible if they provided any HIV prevention, care, treatment, and support services at the health care facility, were at least 18 years of age, spoke English or Swahili, and provided oral consent.

#### Procedures

Trained research assistants used tablets to administer the first two sections of the survey, which asked questions about facility and provider demographic characteristic and PrEP knowledge. Following these sections, research assistants used a standardized, visual script to provide detailed information on PrEP to all HCPs, regardless of their prior PrEP knowledge. To reduce interviewer bias, HCPs were given the tablet to self-administer the remaining survey sections on general perceptions about PrEP and PrEP for AGYW, attitudes and perceptions about adolescent girls’ sexuality, and questions pertaining to the eight domains of quality of care.

### Measures

#### Outcome

The main outcome was willingness to prescribe PrEP to AGYW at high risk of HIV. HCPs were asked to select all that applies to the question “if PrEP became available at this facility today, which of the following sub-populations of AGYW would you provide PrEP if you come in contact with them?” Response options included AGYW in sero-different relationships, who exchange sex for money, who change partners frequently, who had a history of abuse from partners, and who had a history of sexually transmitted infections. Selecting all five of these sub-populations, which has been shown to increase AGYW risk of HIV, was defined as willingness to prescribe PrEP to AGYW at high risk for HIV [1].

#### Covariates

Table 1 presents a description of the quantitative measures used to assess the eight domains of the quality of care framework. An expanded list of quantitative measures can be found in S1 Table. Given the number of measures included, we conducted exploratory factor analysis (EFA) to identify items that could be combined into scales measuring different aspects of the service experience. Measures created using multiple items are considered more reliable than those created from single items, especially when attitudes and opinions are the focus [23]. We used EFA with scree plots and the Kaiser–Meyer–Olkin measure of sampling adequacy to explore the factor structure [24]. We calculated factor loadings to assess item relationships with underlying factors and assessed item uniqueness to ascertain item relevance in the factor model. Items with factors loadings of 0.4 or greater were retained. We computed the Cronbach's alpha statistic for each scale. We created all scales by summing responses to the items; there were no missing responses to the items included in the scales.

**Table 1:** Description of measures used to assess domains of quality of care.

Domain	Quantitative Measures	Qualitative Measures
**Provider-level**		
**Patient-centered care**	**Negative Attitudes Towards Adolescent Sexuality Scale** Alpha: 0.82 Number of items: 11 Possible range: 11–44 Response categories: strongly disagree, disagree, agree, strongly agree Example item: Providing condoms to unmarried adolescent girls (age 15–17) promotes sexual promiscuity **Behavioral Disinhibition Scale** Alpha: 0.79 Number of items: 4 Possible range: 4–19^1^ Response categories: strongly disagree, disagree, neither disagree or agree, agree, strongly agree Example item: The use of PrEP will cause patients to engage in riskier behaviors. **Patient-Centeredness Scale** Alpha: 0.85 Number of Items: 10 Possible Range: 10–40 Response categories: strongly disagree, disagree, agree, strongly agree Example Item: Providers encourage patients to ask questions in order to be responsive to the clients’ needs	In general, how would you describe providers’ attitudes and perceptions towards providing sexual and reproductive health services to adolescent and young adults? [Probe: How do these attitudes and perceptions differ based on: gender, marital status age] What are your opinions about making HIV PrEP available to adolescent girls and young women? [Probe based on age, marital status, sexual behavior]
**Client-staff interactions**	*Questions asked in this domain loaded with the patient-centeredness scale* Example Item: Providers use language clients understand to explain what is happening during the visit.	Would you feel comfortable prescribing PrEP? Why or why not? [Probe: What about to adolescent girls and young women?
**Communication and information**	*Questions asked in this domain loaded with the patient-centeredness scale* Example Item: Providers ensure client privacy and confidentiality by not sharing information without the client’s permission.	How can you support women’s use of PrEP or other daily ARV-based medication? In what ways would you help women understand and manage potential side effects of PrEP?
**Technically competent care**	Do each of seven listed guideline documents (e.g., voluntary counseling and testing guidelines, HIV treatment guidelines) exist at this facility? (*Response categories*: *yes*, *no*, *don’t know)* **Provider Training Adequacy Scale** Alpha: 0.88 Number of items:6 Possible range: 6–24 Response categories: none, some, most, all Example item: Providers have been sufficiently trained to provide HIV prevention and treatment to women	How well do you think this clinic/health facility is currently doing in providing HIV and other sexual/reproductive health services for adolescents? How can service delivery be improved?
**Facility-level**		
**Accessibility**	Are there services available at this facility that specifically target adolescents and young adults? (*Response categories*: *yes*, *no*, *don’t know)*	What do you consider appropriate service-delivery settings to offer PrEP to adolescent girls and young women? Why?
**Efficient and effectively organized care**	On average, how long does a client wait before being seen by a provider (e.g., counselor, nurse, doctor)? (*Response categories*: *less than 15 minutes*, *between 15–30 minutes*, *greater than 30 minutes*) Are there protocols in place to follow-up clients who need additional care? (*Response categories*: *yes*, *no*, *don’t know*) **PrEP Service Impact Scale** Alpha: 0.75 Possible range: 5–25 Response categories: strongly disagree, disagree, neither disagree or agree, agree, strongly agree Example item: I do not have time to provide clinical monitoring required with providing PrEP	In your opinion, what cadre(s) of providers is needed to ensure appropriate counseling, provision, testing, resupply, and follow-up if PrEP was introduced for adolescent girls and young women? Why? What kind of support will health staff need at this facility to provide PrEP? At your clinic/health facility, how would providing PrEP affect existing services?
**Structure and facility**	The waiting rooms are often crowded at this facility. (*Response categories*: *strongly disagree*, *disagree*, *agree*, *strongly agree)*	Where do you think adolescent girls and young women would not feel comfortable accessing services? Why?
**Appropriate package of services**	This facility has experienced stock-outs of 6 listed HIV prevention or treatment options (e.g., condoms, HIV rapid test) in the last 12 months (*Response categories*: *yes*, *no*, *don’t know)* This facility has a system to prevent stock-out of supplies (*Response categories*: *strongly disagree*, *disagree*, *agree*, *strongly agree)*	What kind of sexual and reproductive health services do you provide to adolescents and young adult?

^1^One item in the scale was measured on 4-point scale from strongly disagree to strongly agree

**Provider-level covariates.** We created three scales to capture the patient-centered care domain: the Negative Attitudes Towards Adolescent Sexuality Scale, the Behavioral Disinhibition Scale, and the Patient-Centeredness Scale. The former two scales captured aspects of provider biases and non-stigmatizing care while the latter scale captured whether care is tailored to the needs and preferences of individual clients. We drew some of the items included in the scales from literature [25, 26]. Higher scores on the scales indicated greater negative attitudes toward AGYW’s sexuality, greater concerns that PrEP would result in behavioral disinhibition, and greater provision of patient-centered care, respectively. The survey items designed to measure the quality of care domains of client and staff interactions and communication and information loaded as one factor with the items under the Patient-Centeredness Scale. We created the Provider Training Adequacy Scale to capture the technical competence domain, with higher score indicating that more HCPs perceived they had been adequately trained to provide several HIV-related services. We also assessed the domain based on whether the HCPs had access to the seven service guidelines (e.g., HIV treatment, guidelines). Having the appropriate HIV guidelines was assessed dichotomously, having all seven guidelines or not.

**Facility-level covariates.** We measured the accessibility domain by whether services were available at the facility that focused on adolescents and young adults (“yes” vs. “no/don’t know”). We created the PrEP Service Impact scale to measure the efficiency and effective organization of care domain; higher scores on the scale indicated greater concerns among HCPs about how PrEP introduction would affect current HIV service delivery. We also measured the domain by clients’ waiting time at the facility (less than 15 minutes, between 15 and 30 minutes, greater than 30 minutes) and whether there were protocols in place for client follow-up (yes/no).

We measured the structure and facility domain by HCPs’ report of whether the waiting rooms of the facility were crowded. We dichotomized the responses to “disagree” and “agree” due to low variability. We measured the appropriate package of services domain by whether the facility had experienced at least one stock-out of six HIV prevention or treatment options (e.g., condoms, ARVs) and whether the facility had procedures in place to prevent stock-out of supplies, which was dichotomized to “disagree” and “agree”.

Sociodemographic factors included participant’s age, sex, main profession (nurse, doctor, clinical officer, counselor, other), years worked in profession, number of HIV clients served weekly, prior PrEP knowledge defined as correctly answering nine true/false questions about the medication (e.g., HIV-positive people can take PrEP, oral PrEP does not protect against pregnancy), clinic type (hospital, health center/clinic, dispensary), and facility managing authority (private, public, faith-based organization, military and parastatal).

### Qualitative component

We invited 24 HCPs, 12 per region, who completed the surveys to complete IDIs. HCPs were purposefully selected in order to have perspectives by facility type, management authority, cadre, and gender. Trained research assistants, who were experienced in qualitative interviewing, conducted the IDIs in Swahili with the HCPs.

We developed a semi-structured interview guide in English, which was translated into Swahili then back-translated to ensure correct translation. The guide consisted of questions capturing the eight domains of the quality of care framework, including understanding the sexual and reproductive health services currently provided to adolescents and young adults, attitudes and perceptions toward providing such services, views on PrEP, and PrEP service delivery considerations. Example of questions included in the guide are presented in Table 1. The qualitative codes and their definitions that emerged during analysis for this manuscript are presented S2 Table.

### Analytical approach

Our analyses followed a QUANT-QUAL design and was grounded in the quality of care framework [27]. We designed both the survey and in-depth interview guides to capture information on the domains in the framework (see Table 1). Thus, for this manuscript, we used the framework to distill key aspects of existing services and delivery from the quantitative and qualitative data that would influence providers’ willingness to provide PrEP to AGYW as well as their ability to effectively provide PrEP services. Using the framework, we first analyzed quantitative data. Next, we used the qualitative data analysis results to inform the thematic content analysis of the qualitative data [27]. Specifically, the qualitative data contextualized, further explained, and expanded upon the quantitative findings.

#### Quantitative component

Descriptive statistics were used to describe the outcomes and covariates. Bivariate and multivariable Poisson analyses examined associations among willingness to prescribe PrEP to AGYW at high risk for HIV, domains of quality care, and provider-level characteristics and beliefs. Poisson analyses were applied to calculate relative risks because odds ratios overestimate relative risks when the outcome event was common (incidence of ≥10%) [28]. In the bivariate and multivariate analyses, we used standardized items (mean 0, variance 1) for all the created scales to ensure that they were on the same standard scale for ease of interpretation [29]. We accounted for clustering at the clinic level using Taylor series linearization via survey estimation commands in Stata/SE 13 (StataCorp, College Station, TX).

#### Qualitative component

IDIs were audiotaped, and translated and transcribed verbatim for analysis. Trained research team members verified all transcripts against the original audiotapes to ensure that the transcriptions and translations were accurate. After transcript validation, transcripts were imported into a qualitative software program, ATLAS.ti (Version 8.0) to assist in data analyses.

Study investigators developed the initial coding schemes. Five research team members, led by and including the lead author, coded transcripts. Weekly team meetings were used to discuss and resolve disagreement in coding among coders. When there were no differences in the understanding of and the application of a code, convergent validity was achieved [30]. The lead author reviewed all coding and themes derived from the analyses.

Thematic content analysis was used [31] and themes identifying key factors influencing willingness to PrEP, categorized by the domains of the quality of care framework, were explored and identified by research staff during team meetings. Emphasis was placed on identifying themes that further explained or expanded upon the quantitative results. Coding and identification of themes were an iterative process whereby codes and themes were redefined or merged based on emerging patterns in the data. Following thematic content analysis, a constant comparative method was used to compare themes cross and within regions, facility type, provider type and provider gender [32–34]. The differences, which were minimal, are presented in the domains where they appear.

### Ethical approval

The study protocol was reviewed and approved by the Population Council Institutional Review Board (New York, USA), the National Institute of Medical Research, Medical Research Coordinating Committee (Dar Es Salaam, Tanzania), and the Mbeya Regional Medical Research Ethics Review Committee, (Mbeya, Tanzania). Oral consent, approved by each institutional review board, was obtained from all participants. A research team member signed all consent forms to certify that all consent procedures were followed and received oral consent prior to data collection.

## Results

### Sample description

The characteristics of HCPs and facilities from the quantitative and qualitative components are presented in Table 2. The mean age of the HCPs was 40.3 (standard deviation (sd): 11.6) and they worked in their profession for an average of 13.3 years (sd: 13.3). The majority were female (71.2%) and nurses (48.7%). Most of the sample worked in dispensaries (58.2%), public facilities (60.8%), and facilities located in Dar Es Salaam (62%). Few (3.5%) had prior PrEP knowledge, but 61.1%, once provided with information on PrEP, were willing to prescribe PrEP to AGYW at high risk of HIV.

**Table 2:** Provider and facility characteristics.

	Quantitative Component (n = 316)	Qualitative Component (n = 24)
	N (%) or mean ± sd	N (%) or mean ± sd
**Provider Characteristics**		
Age	40.3 **±** 11.6	41.3 **±** 11.9
Sex		
Male	91 (28.8)	9 (37.5)
Female	225 (71.2)	15 (62.5)
Main profession		
Nurse	154 (48.7)	10 (41.7)
Doctor	48 (15.2)	6 (25.0)
Clinical Officer	39 (12.3)	4 (16.7)
Counselor	41 (13.0)	0 (0.0)
Other (e.g., medical attendant)	34 10.8)	4 (16.7)
Years worked in profession	13.3 **±** 11.9	-
Number of HIV clients served weekly		
0–20	171 (54.1)	-
20 or more	145 (45.9)	-
Prior PrEP knowledge		
No	305 (96.5)	-
Yes	11 (3.5)	-
Willing to prescribe PrEP at AGYW		
No	123 (38.9)	-
Yes	193 (61.1)	-
**Facility Characteristics**		
Regions		
Dar Es Salaam	196 (62.0)	12 (50.0)
Mbeya	120 (38.0)	12 (50.0)
Facility Type		
Hospital	66 (20.9)	6 (25.0)
Health Center	66 (20.9)	9 (37.5)
Dispensary	184 (58.2)	9 (37.5)
Facility Managing Authority		
Private	56 (17.7)	5 (20.8)
Public	192 (60.8)	15 (62.5)
Faith-based organization	42 (13.3)	0 (0.0)
Military	11 (3.5)	3 (12.5)
Parastatal	15 (4.7)	1 (4.2)

The demographic characteristics of the HCPs who completed in-depth interviews were comparable to those who participated in the survey. The mean age of the HCPs was 41.3 (sd: 11.9). The majority were female (62.5%) and nurses (41.7%). An equal proportion (37.5%) of the sample worked in health centers and dispensary, and the majority worked in public facilities (62.5%).

### Provider-level mixed-methods results

#### Patient-centered care domain

The means (sds) for the Negative Attitudes Towards Adolescent Sexuality Scale, the Behavioral Disinhibition Scale, and Patient Centeredness Scale were 19.4 (5.7), 11.0 (4.0), and 34.8 (3.9), respectively (Table 3). Providers from the Dar es Salaam region (p<0.001; 12.0 vs. 10.1) and female providers (19. 9 vs. 18.3; p<0.01) had higher mean scores on the Negative Attitudes Towards Adolescent Sexuality Scale than providers from the Mbeya region and males providers, respectively; scores did not differ by region nor by provider gender on the latter two scales (S3 and S5 Tables). Scale scores did not differ by provider profession (S4 Table). Health centers had lower mean scores than hospitals on the Behavioral Disinhibition Scale (10.1 vs. 12.0; p<0.01) (S6 Table). Facilities managed by the military had higher mean scores than those managed privately on all three scales: Negative Attitudes Towards Adolescent Sexuality Scale (25.4 vs. 21.1; p<0.01), the Behavioral Disinhibition Scale (13.4 vs. 10.9; p<0.01), and Patient Centered Scale (36.7 vs. 34.1; p<0.01) (S7 Table). Faith-based organizations also had higher mean scores on the Patient-Centered Scale than facilities managed privately (35.8 vs. 34.1; p<0.05).

**Table 3:** Quality of care measures (n = 316).

	N (%) or mean ± sd	
**Provider Level**		
**Patient-centered care**		
Negative Attitudes towards Adolescent Sexuality	19.4 **±** 5.7	
Behavioral Disinhibition Scale	11.0 **±** 4.0	
Patient-Centered Scale	34.8 **±** 3.9	
**Technically competent care**		
Provider Training Adequacy Scale	14.2 **±** 3.7	
Has access to HIV guidelines		
No	111 (35.1)	
Yes	205 (64.9)	
**Facility Level**		
**Accessibility**		
Facility has services focused on adolescents & young adults		
No/don't know	63 (19.9)	
Yes	253 (80.1)	
**Efficient and effectively organized care**		
PrEP Service Impact Scale	11.3 **±** 4.1	
Client waiting time at facility		
Less than 15 minutes	124 (39.2)	
Between 15 and 30 minutes	161 (50.9)	
Greater than 30 minutes	31 (9.8)	
Protocols in place for client follow-up		
No	62 (19.6)	
Yes	254 (80.4)	
**Structure and facilities**		
Crowded waiting rooms		
Disagree	166 (52.5)	
Agree	150 (47.5)	
**Appropriate package of services**		
Facility experienced stock-outs of HIV prevention/treatment options in last 12 months		
No	185 (58.5)	
Yes	131 (41.5)	
Facility has system to prevent stockouts of supplies		
Disagree	77 (24.4)	
Agree	239 (75.6)	

In the multivariate analyses, higher scores on the Negative Attitudes Towards Adolescent Sexual Scale and on the Behavioral Disinhibition Scale was associated with lower willingness to prescribe PrEP to AGYW (Adjusted Incidence Rate Ratio (AdjIRR) = 0.81, 95% CI = 0.66–0.99; and AdjIRR = 0.89, 95% CI = 0.79–0.99, respectively) (Table 4). Higher patient-centeredness scores (AdjIRR = 1.19, 95% CI = 0.98–1.45) were marginally associated with higher willingness to prescribe PrEP to AGYW.

**Table 4:** Unadjusted and adjusted incident rate ratios (IRR) for provider willingness to prescribe PrEP for AGYW: Provider-level factors.

	Willingness to Prescribe n/N (%)	Unadjusted IRR (95% CI)	Adjusted IRR^1^ (95% CI)
**Provider Characteristics**			
Age	-	1.01 (1.00–1.01)*	1.01 (0.99–1.02)
Sex			
Male	59/91 (64.8)	1.00	1.00
Female	134/225 (59.6)	0.92 (0.78–1.09)	0.88 (0.72–1.08)
Main profession			
Nurse	96/154 (62.3)	1.00	1.00
Doctor	31/48 (64.6)	1.04 (0.82–1.31)	1.01 (0.81–1.28)
Clinical Officer	26/39 (66.7)	1.07 (0.85–1.34)	0.89 (0.68–1.17)
Counselor	24/41 (58.5)	0.94 (0.67–1.31)	1.01 (0.74–1.37)
Other (e.g., medical attendant)	16/34 (47.1)	0.75 (0.50–1.13)	0.75 (0.52–1.08)
Years worked in profession	-	1.01 (1.00–1.01)*	1.00 (0.99–1.02)
Prior PrEP knowledge			
No	185/305 (60.7)	1.00	1.00
Yes	8/11 (72.7)	1.20 (0.80–1.80)	1.23 (0.86–1.78)
Number of HIV clients served weekly			
0–20	103/171 (60.2)	1.00	1.00
20 or more	90/145 (62.1)	1.03 (0.88–1.21)	0.98 (0.82–1.16)
**Quality of Care Measures**			
**Patient-centered care**			
Negative Attitudes towards Adolescent Sexuality	-	0.68 (0.58–0.81)**	0.81 (0.66–0.99)*
Behavioral Disinhibition Scale	-	0.81 (0.72–0.92)**	0.89 (0.79–0.99)*
Patient-Centered Scale	-	1.32 (1.10–1.59)**	1.19 (0.98–1.45)^+^
**Technically competent care**			
Provider Training Adequacy Scale	-	1.18 (1.06–1.30)**	1.13 (1.02–1.24)*
Has access to HIV guidelines			
No	63/111 (56.8)	1.00	1.00
Yes	130/205 (63.4)	1.12 (0.91–1.37)	0.98 (0.79–1.21)

^+^ <0.10

* <0.05

**<0.01

^1^ Adjusted for all facility-level measures: region, facility type, facility managing authority, services for adolescents, PrEP Service Impact Scale, client waiting time, protocols for client follow-up, crowded waiting rooms, stockouts of prevention and treatment options and system to prevent stockouts

**Theme 1.** Stigma and discrimination toward adolescents: A majority of HCPs described clinical environments where adolescents are often embarrassed, criticized, and judged by colleagues. Providers described witnessing instances where unmarried adolescents are ignored, not provided enough attention as required for their healthcare need, and are rebuked for seeking sexual and reproductive health services. In contrast, married AGYW are welcomed and receive good service. HCPs explained that one of the primary reasons for such treatment of adolescents is Tanzanian culture and traditions that discourage young girls from having sex before marriage. Those who engage in pre-marital sex are often stigmatized by their communities, including HCPs, as being promiscuous. These cultural beliefs result in some HCPs being apprehensive about providing sexual and reproductive health care (e.g., contraceptive) to AGYW; some HCPs noted the same can happen with PrEP introduction.

Some of the health providers when they see a young girl of about 16 or 17 years old, they start mocking her by saying that how come such a young girl has come to seek family planning health services! Does she have a husband or has she started sexual activities? That will make the client disappear from that clinic completely …some health providers have these challenges of discouraging clients. (Female nurse, age 62, private health center)

For those who are not married, there is a bit of stigma to some of them. Some of the service providers have stigma…because it the culture of our society that when someone talks about contraceptives she should be married, if not married she is a strange person. Therefore, customs and traditions enhance the stigma as well. (Male doctor, age 57, public health center)

Theme 2. Concerns about behavioral disinhibition due to PrEP: Some HCPs expressed apprehension toward PrEP as they feared its use will result in users neglecting previously protective behaviors, such as condom use, resulting in increased rates of STIs and unintended pregnancies. A few HCPs were concerned about adolescents and young adults becoming sexually active due to PrEP.

I have doubts on the availability of PrEP. People may become careless and get involved in sexual activities knowing that there is a preventive measure for HIV. People had been living with fear of getting HIV infection and they were using condoms. Also, many had refrained from sex; but they will revert to bad habits. I think there would be an increase of other sexual transmitted infections as most people do not fear syphilis or gonorrhea. (Male clinical officer, age 32, public hospital)

On the other hand, some HCPs disagreed with the notion of behavioral disinhibition, explaining that even with the fear of HIV, individuals are still engaging in unprotected sexual intercourse and having multiple partners, as evidenced by the high rates of HIV.

Others think that if you give this [referring to PrEP] it may increase risk behavior but personally I don't think so. Bad behaviors are still existing even after HIV, so it’s like this you have decided to help people (Female nurse, age 26, public dispensary)

#### Client and staff interactions domain

Lack of confidentiality around adolescent care: HCPs described clinical environments in which there is a lack of respect and confidentiality when providing care to adolescents, some HCPs are inattentive to adolescents, and some HCPs require parents to be present before providing services to adolescents. The latter issue was prevalent in the discussions with Mbeya HCPs but not Dar es Salaam HCPs. These were key barriers to adolescents seeking sexual and reproductive health care, and therefore, can impact PrEP service provision.

There is different perception. Even the customers are aware of that. When for example a young teenager comes to the dispensary and find out that I am on duty [pause] they normally ask first “who is there today?” “It’s Mr. so and so; Ooh that one will give to you”; “and what about today? “There is a certain woman; Ooh that one no, after all she will tell your mother about it”. So if customers knows that we give out services differently. (Male assistant clinical officer, age 32, public dispensary)

#### Communication and information domain

Provider provision of patient education key for PrEP use and adherence: Despite the barriers to PrEP noted by HCPs, most HCPs who participated in IDIs were willing to support AGYW’s use of PrEP. Education was the consistent answer to how they could offer support to AGYW who wanted to take PrEP. HCPs described that comprehensive education provided on a consistent basis in a welcoming and friendly environment was key to encouraging AGYW to use and adhere to PrEP. This was especially the case when discussing behavioral disinhibition. Regardless of the HCPs beliefs surrounding behavioral disinhibition, most explained appropriate education and counseling to clients was key to avoiding behavioral disinhibition.

You must keep reiterating that knowledge every time when she comes to the clinic. Don’t say that I have already told her the importance of using the medications and therefore when she comes the second time you just say, “I told her about this last time, or if I keep telling her; she will get tired.” You can do it even in different words but the aim is to insist on the adherence of the medications so that she can benefit from that medicine. Therefore, that will encourage her. (Male doctor, age 57, public health center)

They should be given enough education on proper use of PrEP. A full package education and not a partial one, making sure they understand and you ask them some questions on their understandings on what you were teaching. (Female nurse, age 34, public dispensary)

#### Technically competent care domain

The mean (sd) for the Provider Training Adequacy Scale was 14.2 (sd: 3.7) (Table 3), with health centers (13.6 vs. 14.9; p<0.05) and doctors (13.3 vs. 14.5; p<0.05) having significantly lower mean scores in comparison to hospitals and nurses, respectively (S4 and S6 Tables). In the multivariate analyses, higher scores on Provider Training Adequacy Scale were associated with higher willingness to prescribe PrEP to AGYW (AdjIRR = 1.13, 95%CI = 1.02–1.24) (Table 4).

Adequate and regular training to provide PrEP: Providers expressed a need for training to provide adolescent-friendly care and regular training to provide optimal HIV prevention and treatment services. They emphasized the need for on-the-job training over off-site training seminars, especially training on how to provide HIV counseling. They also requested such resources as information, educational, and communication materials, and pocket-size reference tools that can supplement the training received. These will be important to ensuring they are able to provide quality PrEP services to adolescents.

Yes, we will need training because this youth friendly service is a kind of service which needs a long-time training and cannot be given in a single day and think that you’re capable of providing services effectively. This is especially in my center, we will need training on youth friendly services because up to now only one service provider got a chance to attend a one-day training but I think responsible staff are working on it. (Male medical assistant, age 31, public health center)

*Expertise is required on testing* [and] *counseling since these go along side with giving out the PrEP medicine*. *As you know*, *the staff need to be updated frequently*. *Although they are all trained personnel*, *some may neglect a few things and they need to get a refresher course training in certain areas to be aware of new things*. *(Male clinical officer*, *age 27*, *public dispensary)*

### Facility-level mixed-methods results

#### Accessibility domain

Although 80.1% of HCPs reported that their facility had services specifically for adolescents and young adults (Table 3), a larger proportion of male providers disagreed such services existed as compared to female providers (28.6% vs. 16.4%; p<0.05) (S3 Table). A larger proportion of HCPs (63.8%) who reported that their facility had services focused on adolescents and young adults were willing to prescribe PrEP to AGYW than those who reported their facility did not have such services (54%) (Table 5). However, the difference was not significant in multivariate analyses.

**Table 5:** Unadjusted and adjusted incident rate ratios (IRR) for provider willingness to prescribe PrEP for AGYW: Facility-level factors.

	Willingness to Prescribe n/N (%)	Unadjusted IRR (95% CI)	Adjusted IRR^1^ (95% CI)
**Facility Characteristics**			
**District**			
Dar Es Salaam	115/198 (58.6)	1.00	1.00
Mbeya	78/120 (65.0)	1.11 (0.92–1.33)	1.08 (0.91–1.30)
**Facility type**			
Hospital	37/66 (56.1)	1.00	1.00
Health center	39/66 (59.1)	1.05 (0.82–1.35)	1.12 (0.89–1.41)
Dispensary	117/184 (63.6)	1.13 (0.91–1.41)	1.26 (1.05–1.52)*
**Facility managing authority**			
Private	33/56 (58.9)	1.00	1.00
Public	123/192 (64.1)	1.09 (0.84–1.40)	0.86 (0.68–1.10)
Faith-based organization	24/42 (57.1)	0.97 (0.71–1.33)	0.77 (0.58–1.03)^+^
Military	5/11 (45.5)	0.77 (0.60–0.99)*	0.86 (0.65–1.15)
Parastatal	8/15 (53.3)	0.91 (0.54–1.51)	0.65 (0.38–1.12)
**Quality of Care Measures**			
**Accessibility**			
Facility has services focused on adolescents and young adults			
No/don't know	34/63 (54.0)	1.00	1.00
Yes	159/253 (62.8)	1.16 (0.90–1.51)	1.02 (0.78–1.33)
**Efficient and effectively organized care**			
PrEP Service Impact Scale	—	0.79 (0.68–0.90)**	0.96 (0.82–1.12)
Client waiting time at facility			
Less than 15 minutes	69/124 (55.6)	1.00	1.00
Between 15–30 minutes	101/161 (62.7)	1.13 (0.91–1.39)	1.09 (0.89–1.33)
Greater than 30 minutes	23/31 (74.2)	1.33 (1.01–1.75)*	1.35 (1.04–1.76)*
Protocols in place for client follow-up			
No	35/62 (56.5)	1.00	1.00
Yes	158/254 (62.2)	1.10 (0.87–1.39)	0.95 (0.76–1.19)
**Structure and facilities**			
Crowded waiting rooms			
Disagree	93/166 (56)	1.00	1.00
Agree	100/150 (66.7)	1.19 (0.97–1.45)+	1.18 (0.95–1.46)
**Appropriate package of services**			
Facility had stockouts of HIV prevention and treatment options in last 12 months			
No	111/185 (60.0)	1.00	1.00
Yes	82/131 (62.6)	1.04 (0.85–1.28)	0.99 (0.81–1.20)
Facility has system to prevent stockouts of supplies			
Disagree	46/77 (59.7)	1.00	1.00
Agree	147/239 (61.5)	1.03 (0.86–1.24)	1.05 (0.84–1.31)

^+^ <0.10

* <0.05

**<0.01

^1^Adjusted for all provider-level measures: age, gender, main profession, years worked in profession, prior PrEP knowledge, number of PLHIV served, Negative Attitudes Towards Adolescent Sexuality Scale, Behavioral Disinhibition Scale, Provider Training Adequacy Scale, Patient-Centered Care Scale, and had access to HIV guidelines

#### Efficient and effectively organized care domain

The mean (sd) for the PrEP Impact Scale was 11.3 (4.1); providers from the Dar es Salaam region (12.0 vs. 10.1; p<0.01) and female providers (11.7 vs. 10.3; p<0.01) had higher scores on the scale than providers from the Mbeya region and male providers, respectively.

Higher scores on the PrEP Service Impact Scale was significantly associated with reduced willingness to prescribe PrEP to AGYW in the bivariate analyses but not in multivariate analyses (Table 5). Compared to HCPs who reported that the waiting time at their facility was less than 15 minutes, those who reported it was greater than 30 minutes were more willing to prescribe PrEP to AGYW (AdjIRR = 1.36, 95%CI = 1.04–1.79).

Negative impact of PrEP on existing services: HCPs expressed many concerns about how PrEP will affect the care they provide to clients. Contrary to the quantitative findings, HCPs felt that long waits would hinder PrEP access, a noted barrier to existing sexual and reproductive health services. HCPs theorized that should PrEP result in an influx of clients requesting the medication, there would be a shortage of staff to provide timely care. Some noted that staff shortages already exist. Staff shortages will have a ripple effect whereby their individual workload would increase, leading to clinic congestion. Some HCPs expressed the need for additional space to provide PrEP services. HCPs in Mbeya and those working dispensaries expressed concerns about not having the appropriate equipment to monitor the major side effects of PrEP. HCPs were also concerned about the potential for PrEP drug shortages and whether an adequate supply can be maintained. Should staff shortages increase or drug shortages occur, HCPs feared that these will result in low uptake and poor adherence to PrEP as clients would be turned-off by these barriers.

Shortage of staff is what will make people think much about coming to clinic either for 3 months checkup or to get drugs and when they come and wait for so long they probably won't like it so staff, resources should be provided. (Female nurse, age 34, public dispensary)

To reduce the potential negative impact of PrEP on existing services, HCPs in the Mbeya region recommended leveraging the use of paramedical professionals (e.g., community-based health workers) to provide PrEP services to AGYW. Paramedical professionals, in their view, can be trained to provide the services and closely monitor adherence. Moreover, paramedical professionals have close community relations and connections, whereby the community accepts and trusts them. These strengths can facilitate increase uptake of PrEP.

The cadres are already known [to provide PrEP]. These are doctors, nurses, assistant doctors and nurses; there are also medical attendants who assist. We also have a group of HBCs; if given appropriate knowledge, they can provide services. [HBCs] are very close to the community. The community will always accept them. The HBCs have a very good client relationship and they assist other cadres. (Male clinical officer, age 32, public hospital)

#### Structure and facilities domain

Almost half (47.5%) of HCPs agreed that their waiting rooms were crowded (Table 3). HCPs’ perceptions of the crowdedness of the waiting rooms at their facility was not associated with their willingness to prescribe PrEP to AGYW (Table 5).

Integration of PrEP into comprehensive services reduces PrEP stigma: When asked about where PrEP should be provided to foster the uptake of PrEP by adolescents and young adults, HCPs focused on the importance of PrEP integration into comprehensive services, the use of different types of health care institutions, and the use of different types of health care personnel, such as paramedical professionals. Underscoring these recommendations was the need for privacy and confidentiality. HCPs were familiar with ARVs; thus, they easily understood PrEP and the potential issues that would be associated with its introduction. If PrEP was offered only by public health facilities providing HIV services, specifically those distributing ARVs, HCPs feared that the prophylaxis would be stigmatized, thus deterring clients, especially adolescents and young adults, from accessing it. Additionally, when offered in different health care institutions (e.g., public, private, community-based organizations), HCPs envisioned the burden experienced by public health staff would be considerably reduced and staff shortages less likely.

The best environment [to provide PrEP] should be multi-sectoral…. The government institutions should not provide these services alone because the private institutions have very good doctors like those found in government institutions…. We have home-based care whom we can use to provide these services…because they deal with HIV patients at homes, by assisting them to easily access the ARV medicines, though they need more education. The mobile clinics can be used if they have experts. As you know, girls and young women tend to shy off in public clinics which are involved in providing HIV services; therefore, this will provide more privacy…. The girls and young females would always avoid the stigma of being associated with HIV infection. (Male clinical officer, age 32, public hospital)

Locations where girls and women will feel embarrassed to go for PrEP are clinics where ARVs is being distributed. We will not be able to get the clients…This needs to be made private. It may be better to place this service under family planning clinic. (Female doctor, age 55, public health center)

#### Appropriate package of services domain

A total of 41.5% of HCPs reported that their facility had experienced stock-outs of at least one HIV prevention and treatment option in the last 12 months but 75.6% noted there was a system to prevent stock-outs of supplies (Table 3). Mbeya, however, experienced greater stock-outs of HIV preventive and treatment options than Dar es Salaam (20.6% vs. 17.4%, p<0.05) (S5 Table).

Experiencing stock-outs of HIV prevention and treatment options and having a system to prevent stock-outs of supplies were not significantly associated with willingness to provide PrEP to AGYW (Table 5).

Adolescent need for sexual and reproductive services: Thi**s** emerged as an important reason for HCPs’ willingness to provide PrEP to AGYW. Some HCPs described circumstances that made AGYW especially vulnerable to HIV. HCPs noted high rates of sexual violence (e.g., rape, coercion) against both their married and unmarried AGYW clients. Others expressed that their sexually active AGYW clients engage in high risk behaviors. Therefore, regardless of their own personal beliefs, AGYW have a right to sexual and reproductive health care and need a prevention method that works. As such, PrEP should be included in the prevention options offered to AGYW.

This young generation will benefit because they are in a risky environment, traumatized, raped and forced to get married when they are very young. The use of PrEP will prevention from getting HIV infection. (Male clinical officer, age 27, public dispensary)

She may say, My husband doesn’t care about me, he beats me every day and he moves out carelessly and I am afraid he can infect me with HIV….” You may test her negative but the problem of being raped at home is still there, but now you can give a solution that now you can use PrEP and the remaining problem will be being beaten but HIV infections will not be there. (Male doctor, age 57, public health center)

## Discussion

The use of oral PrEP can aid in achieving HIV epidemic control among AGYW in sub-Saharan Africa, where high HIV incidence rates continue to be troublingly high among AGYW. Understanding how to effectively introduce and deliver PrEP to AGYW to ensure they can access, use, and adhere to it is critically important to reducing incidence among AGYW. HCPs are crucial for the success of PrEP. To our knowledge, we conducted the first mixed-methods study assessing HCPs’ willingness to prescribe PrEP to AGYW in a sub-Saharan African setting. Overall, a large proportion of HCPs were willing to prescribe PrEP to AGYW. However, it is insufficient to train HCPs on solely the clinical aspects of providing PrEP. We identified key barriers to HCPs’ provision of PrEP to AGYW as well as key opportunities for HCPs to make PrEP accessible and available to AGYW. Study findings call for the use of a quality of care framework to immediately implement strategies that: 1) reduce HCPs’ biases toward providing sexual and reproductive health services to AGYW; and 2) adequately prepare the health system infrastructure for the introduction of PrEP.

HCPs’ biases toward AGYW’s sexuality, which are grounded in cultural norms, often result in stigmatizing and discriminatory care experienced by AGYW. These biases can negatively impact AGYW’s access and use of PrEP, whereby HCPs who hold these attitudes and beliefs will be less likely to provide PrEP to AGYW and AGYW will be less likely to access PrEP services based on existing poor services. A qualitative study among HCPs in Kenya found that discrimination and stigma by staff toward at-risk clients poses a significant challenge to PrEP implementation [35]. To overcome these challenges, HCPs should be provided with regular training to provide high-quality, patient-centered, and youth-friendly services as highlighted by both the quantitative and qualitative findings. Additionally, appropriate counseling strategies and tools are needed for HCP use given the important role patient education will play in PrEP uptake and adherence. Regularly monitoring service delivery can identify when refresher trainings are warranted. Existing studies conducted in the United States and Latin America have also noted that lack of training of HCPs as a main barrier to PrEP provision [36–40]. A critical aspect of the training should center on stigma reduction and values clarification. A study in Bangladesh found that a short, targeted stigma reduction intervention among HCPs that included sessions focused on reflecting on personal values and negative impacts of stigma, rapidly improved provider attitudes and increased service satisfaction among young people [17]. Similar interventions are needed during the preparatory stages of PrEP implementation in Tanzania and other sub-Saharan African countries. HCPs should be involved in the development or adaption of training materials to ensure the training is applicable to their clinical settings.

Training and PrEP implementation strategies should address HCPs’ concerns around behavioral disinhibition given its potential role in influencing their prescribing behaviors toward AGYW. Our quantitative findings showed that behavioral disinhibition concerns were significantly associated with HCPs’ lower willingness to provide PrEP to AGYW, but our qualitative findings were mixed with some HCPs having concerns about behavioral disinhibition and others not. Concerns about behavioral disinhibition remain a consistent barrier to PrEP provision among HCPs in other studies despite several PrEP trials reporting they have not found behavioral disinhibition or increase sexually transmitted diseases after PrEP implementation [40–44]. Therefore, it is important to continuously evaluate whether these fears are hindering AGYW access to PrEP after implementation.

To provide PrEP as a part of a comprehensive prevention package as recommended by WHO, the health system must be adequately prepared. HCPs in our study confirmed the need to be prepared to take on PrEP as an additional health service. They recommended the use of paramedical professionals and integrating PrEP into services beyond HIV as key PrEP implementation strategies. Other researchers have suggested task-shifting/sharing within the health system as an effective PrEP implementation strategy [35], but the use of paramedical professionals goes beyond task-shifting to focus on a community-based approach to PrEP introduction. Paramedical professionals may also alleviate HCPs’ concerns about PrEP introduction stretching an already overburdened and short-staffed health system, concerns that have been reported by other studies [35, 39, 40]. The use of paramedical professionals coupled with PrEP integration into services beyond HIV, could also reduce potential stigma and discrimination experienced by AGYW when accessing these services. However, additional implementation science studies are needed to evaluate how these professionals can be used in increasing access to and support adherence to PrEP, especially among AGYW, and how integration can be accomplished in settings like Tanzania where ARV services are otherwise confined to specific HIV care and treatment centers dedicated to serving people living with HIV.

The finding that longer waiting times is associated with willingness to prescribe PrEP could be reflective of the amount of time clients are spending in a consultation room with HCPs. That is, those HCPs would be more willing to provide PrEP after spending more time with clients to gather a comprehensive medical history and to counsel clients. Some evidence supporting this is that a significantly higher proportion of providers who disagreed with the statement “providers have sufficient time to talk to each client about their medical history” reported that client wait times were greater than 30 minutes (23% vs. 8%). This longer length of time attending to each patient does, however, result in longer waiting times when there are large client volumes.

With regards to the concerns about drug shortages, there is a need to conduct logistics and modeling studies to project for long-term procurement of PrEP. Recent findings from a phase 2 clinical trial among South African women show that in comparison to time-driven or event-driven PrEP dosing, daily use of PrEP resulted in protection over a greater number of sex acts and greater adherence [45]. Therefore, drug shortages can hinder both access and adherence among AGYW. There is also need for studies on how adherence can be supported for time and event-driven PrEP dosing rather than daily PrEP use among women as a potential avenue of reducing the costs to the healthcare system.

### Methodological considerations

First, PrEP is not yet available in the health system in Tanzania and therefore, it cannot be said with certainty that HCPs’ reports in our study reflects how they will actually behave if and when the intervention becomes available nor can we say with certainty what issues will exist related to drug supply. However, HCPs are familiar with ARVs and post-exposure prophylaxis; thus, they easily understood PrEP and the circumstances associated with its introduction. Second, self-reported clinical practices and perceptions of service quality may not accurately reflect the clinical environment. However, the use of both qualitative and quantitative methods allows for the triangulation of findings and contributes to making our findings more robust. Our sample was representative of health facilities in the two selected councils, allowing for generalizability of findings.

## Conclusions

In summary, our findings highlight key considerations that should be addressed when preparing HCPs to provide PrEP generally and to AGYW, specifically. It is important that comprehensive and regular on-the-job training that incorporates sensitization on caring and working with adolescents and young adults, especially AGYW, and that addresses HCPs’ biases be a part of PrEP service provision training. The use of a quality of care framework in the development of such trainings can ensure that all appropriate domains of care are adequately covered. There is also a need to clearly evaluate how PrEP introduction might impact the delivery of services in the current health system and assess the feasibility of an integrated approach to its delivery. It is essential that the necessary preparatory steps are in place to ensure that AGYW can access, use, and adhere PrEP.

## Supporting information

S1 Table Quantitative measures. (DOCX)

S2 Table Qualitative measures. (DOCX)

S3 Table Quality of care measures by provider sex. (DOCX)

S4 Table Quality of care measures by provider occupation. (DOCX)

S5 Table Quality of care measures by region. (DOCX)

S6 Table Quality of care measures by facility type. (DOCX)

S7 Table Quality of care measures by facility management. (DOCX)
