# Supplementary material for: Provider perspectives on PrEP for adolescent girls and young women in Tanzania: The role of provider biases and quality of care
Source: PLoS One. 2018 Apr 27;13(4):e0196280. doi: 10.1371/journal.pone.0196280 (PMC5922529; doi:10.1371/journal.pone.0196280)
Supplement: S1 Table — (DOCX) [file pone.0196280.s001.docx]

**Supporting Information 1**

| **Quantitative Questions Used to Assess Domains of Quality of Care, the Outcome and Demographic Characteristics** | | | | |
| --- | --- | --- | --- | --- |
| **English** | | | **Swahili** | |
| **PROVIDER-LEVEL** | | | | |
| **DOMAIN: Patient-centered care** | | | | |
| ***Negative Attitudes Towards Adolescent Sexuality Scale Items*** | | | | |
| I am uncomfortable providing sexual and reproductive health services to |  | Sijisikii vizuri kutoa huduma za afya ya uzazi kwa; | |  |
| adolescent girls (age 15-17) | 1. Strongly disagree 2. Disagree 3. Agree 4. Strongly agree | Mabinti wenye umri wa miaka 15-17 | | 1. Sikubaliani kabisa 2. Sikubaliani 3. Nakubaliana 4. Nakubaliana Kabisa |
| young women (age 18-24) | 1. Strongly disagree 2. Disagree 3. Agree 4. Strongly agree | Wanawake wenye umri wa miaka 18-24 | | 1. Sikubaliani kabisa 2. Sikubaliani 3. Nakubaliana 4. Nakubaliana Kabisa |
| Providing condoms to following persons promotes sexual promiscuity |  | Kugawa kondomu watu wafuatao kunakuza uasherati; | |  |
| unmarried adolescent girls (age 15-17) | 1. Strongly disagree 2. Disagree 3. Agree 4. Strongly agree | Mabinti wadogo wasioolewa wenye umri wa miaka 15-17 | | 1. Sikubaliani kabisa 2. Sikubaliani 3. Nakubaliana 4. Nakubaliana Kabisa |
| unmarried young women (age 18-24) | 1. Strongly disagree 2. Disagree 3. Agree 4. Strongly agree | Wanawake wadogo wasioolewa wenye umri wa miaka 18-24 | | 1. Sikubaliani kabisa 2. Sikubaliani 3. Nakubaliana 4. Nakubaliana Kabisa |
| Unmarried adolescent girls should not be provided with contraceptives because Tanzanian culture does not support premarital sex. | 1. Strongly disagree 2. Disagree 3. Agree 4. Strongly agree | Vijana wa kike ambao hawajaolewa hawatakiwi wapewe njia za uzazi wa mpango kwa sababu mila za kitanzania haziruhusu kufanya mapenzi kabla ya ndoa. | | 1. Sikubaliani kabisa 2. Sikubaliani 3. Nakubaliana 4. Nakubaliana Kabisa |
| It is better to tell the following persons to abstain from sex when they ask for condoms rather than giving them condoms |  | Ni bora kuwaambia makundi yafuatayo kuacha kujamiiana pale ambapo wataomba kondomu badala ya kuwapa kondomu hizo; | |  |
| adolescent girls (age 15-17) | 1. Strongly disagree 2. Disagree 3. Agree 4. Strongly agree | Mabinti wenye umri 15-17 | | 1. Sikubaliani kabisa 2. Sikubaliani 3. Nakubaliana 4. Nakubaliana Kabisa |
| young women (age 18-24) | 1. Strongly disagree 2. Disagree 3. Agree 4. Strongly agree | Wanawake wadogo wenye umri 18-24 | | 1. Sikubaliani kabisa 2. Sikubaliani 3. Nakubaliana 4. Nakubaliana Kabisa |
| Adolescent girls age 15-17: |  | Vijana wa kike wenye miaka 15-17; | |  |
| should be ashamed to ask their provider for PrEP | 1. Strongly disagree 2. Disagree 3. Agree 4. Strongly agree | Wanatakiwa waone haya kuomba PrEP kutoka kwa mtoa huduma wao | | 1. Sikubaliani kabisa 2. Sikubaliani 3. Nakubaliana 4. Nakubaliana Kabisa |
| should not be given PrEP because they should not be having sex | 1. Strongly disagree 2. Disagree 3. Agree 4. Strongly agree | Hawatakiwi kupewa PrEP kwa kuwa hawataki kufanya ngono | | 1. Sikubaliani kabisa 2. Sikubaliani 3. Nakubaliana 4. Nakubaliana Kabisa |
| Young women age 18-24: |  | Wanawake wenye umri mdogo wa miaka 18-24; | | 1. Sikubaliani kabisa 2. Sikubaliani 3. Nakubaliana 4. Nakubaliana Kabisa |
| should be ashamed to ask their provider for PrEP | 1. Strongly disagree 2. Disagree 3. Agree 4. Strongly agree | Wanatakiwa waone haya kuomba PrEP kutoka kwa mtoa huduma wao | | 1. Sikubaliani kabisa 2. Sikubaliani 3. Nakubaliana 4. Nakubaliana Kabisa |
| should not be given PrEP because they should not be having sex | 1. Strongly disagree 2. Disagree 3. Agree 4. Strongly agree | Hawatakiwi kupewa PrEP kwa kuwa hawataki kufanya ngono | | 1. Sikubaliani kabisa 2. Sikubaliani 3. Nakubaliana 4. Nakubaliana Kabisa |
| ***Behavioral Disinhibition Scale Items*** | | | | |
| The use of PrEP will cause patients to engage in riskier behaviors. | 1. Strongly disagree  2. Disagree  3. Neither agree nor disagree  4. Agree  5. Strongly agree | Matumizi ya PrEP yatasababisha wagonjwa wajihusishe zaidi kwenye tabia hatarishi. | | 1. Sikubaliani kabisa 2. Sikubaliani 3. Siko upande wowote  4. Nakubaliana 5. Nakubaliana Kabisa |
| I believe that providing PrEP endorses the risk behaviors of users | 1. Strongly disagree  2. Disagree  3. Neither agree nor disagree  4. Agree  5. Strongly agree | Ninaamini kwamba toaji wa PrEP unaidhinisha tabia hatarishi za watumiaji | | 1. Sikubaliani kabisa 2. Sikubaliani 3. Siko upande wowote  4. Nakubaliana 5. Nakubaliana Kabisa |
| Providing PrEP will result in an increase in sexually transmitted diseases among patients | 1. Strongly disagree  2. Disagree  3. Neither agree nor disagree  4. Agree  5. Strongly agree | Utoaji wa PrEP utasababisha ongezeko la magonjwa ya zinaa miongoni mwa wagonjwa. | | 1. Sikubaliani kabisa 2. Sikubaliani 3. Siko upande wowote  4. Nakubaliana 5. Nakubaliana Kabisa |
| The use of PrEP will cause adolescent girls and young women to engage in riskier sexual behaviors | 1. Strongly disagree 2. Disagree 3. Agree 4. Strongly agree | Matumizi ya PrEP yatasababisha vijana wa kike na wanawake wenye umri mdogo wajihusishe na tabia hatarishi zaidi | | 1. Sikubaliani kabisa 2. Sikubaliani 3. Nakubaliana 4. Nakubaliana Kabisa |
| ***Patient-Centeredness Scale Items*** | | | | |
| Providers encourage patients to ask questions in order to be responsive to the clients’ needs | 1. Strongly disagree 2. Disagree 3. Agree 4. Strongly agree | Watoa huduma huwahamasisha wagonjwa kuuliza maswali ili watoe huduma zinazoendana na mahitaji ya wagonjwa | | 1. Sikubaliani kabisa 2. Sikubaliani 3. Nakubaliana 4. Nakubaliana Kabisa |
| Providers ensure client privacy and confidentiality by not sharing information without the client’s permission^1^ | 1. Strongly disagree 2. Disagree 3. Agree 4. Strongly agree | Watoa huduma huhakikisha faragha na usiri kwa kutokutoa taatifa za mteja bila ruhusa ya mteja. | | 1. Sikubaliani kabisa 2. Sikubaliani 3. Nakubaliana 4. Nakubaliana Kabisa |
| Providers make sure to speak with adolescent clients privately (without parents or other people in the room).^1^ | 1. Strongly disagree 2. Disagree 3. Agree 4. Strongly agree | Watoa huduma wanahakikisha kuwa wanazungumza na wateja vijana kwa faragha (bila wazazi au watu wengine kuwepo katika chumba) | | 1. Sikubaliani kabisa 2. Sikubaliani 3. Nakubaliana 4. Nakubaliana Kabisa |
| Providers take the time to understand individual client needs and circumstances | 1. Strongly disagree 2. Disagree 3. Agree 4. Strongly agree | Watoa huduma hutumia muda ili kuelewa mahitaji binafsi na hali ya kila mteja | | 1. Sikubaliani kabisa 2. Sikubaliani 3. Nakubaliana 4. Nakubaliana Kabisa |
| Providers counsel clients about HIV prevention options (e.g. male and female condoms) available to them | 1. Strongly disagree 2. Disagree 3. Agree 4. Strongly agree | Watoa huduma hushauri wateja kuhusu njia za kuzuia maambukizi ya VVU zinazopatikana kwa ajili yao (mfano kondomu za kike na za kimue) | | 1. Sikubaliani kabisa 2. Sikubaliani 3. Nakubaliana 4. Nakubaliana Kabisa |
| Providers assess clients’ knowledge gaps in clients about HIV prevention and treatment in order to determine the level of information to provide^2^ | 1. Strongly disagree 2. Disagree 3. Agree 4. Strongly agree | Watoa huduma hutathmini mapungufu kwenye ufahamu wa wateja kuhusu kinga na matibabu ya VVU ili kuamua kiwango cha taarifa za kuwapatia. | | 1. Sikubaliani kabisa 2. Sikubaliani 3. Nakubaliana 4. Nakubaliana Kabisa |
| Providers provide the necessary information of HIV prevention options, including instructions on use and follow-up required | 1. Strongly disagree 2. Disagree 3. Agree 4. Strongly agree | Watoa huduma hutoa taarifa muhimu kuhusu namna za kujikinga na VVU, ikijumuishwa na maelekezo ya matumizi na ufuatiliaji unaotakiwa | | 1. Sikubaliani kabisa 2. Sikubaliani 3. Nakubaliana 4. Nakubaliana Kabisa |
| Providers counsel clients about HIV testing and repeat testing. | 1. Strongly disagree 2. Disagree 3. Agree 4. Strongly agree | Watoa huduma hushauri wateja kuhusu upimaji na kurudia kupima. | | 1. Sikubaliani kabisa 2. Sikubaliani 3. Nakubaliana 4. Nakubaliana Kabisa |
| Providers treat adolescents and young adults with dignity and respect^1^ | 1. Strongly disagree 2. Disagree 3. Agree 4. Strongly agree | Watoa huduma huwahudumia vijana kwa kuwaheshimu na staha. | | 1. Sikubaliani kabisa 2. Sikubaliani 3. Nakubaliana 4. Nakubaliana Kabisa |
| Providers use language clients understand to explain what is happening during the visit.^2^ | 1. Strongly disagree 2. Disagree 3. Agree 4. Strongly agree | Watoa huduma wanatumia lugha ambayo wateja wanaielewa katika kueleza kile kinachotokea wakati wa ziara. | | 1. Sikubaliani kabisa 2. Sikubaliani 3. Nakubaliana 4. Nakubaliana Kabisa |
| **Technically competent care** |  |  | |  |
| Do the following documents (e.g., guideline, manual) exist at this facility? |  | Je, nyaraka zifuatazo (kwa mfano, miongozo) zipo katika kituo hiki? | |  |
| Voluntary counseling and testing guidelines | 1. Yes  2. No  3. Don’t Know | Miongozo ya ushauri nasaha na upimaji wa hiari | | 1. Ndiyo  2. Hapana  3. Sifahamu |
| HIV treatment guidelines | 1. Yes  2. No  3. Don’t Know | Miongozo ya tiba ya VVU | | 1. Ndiyo  2. Hapana  3. Sifahamu |
| STI management and treatment guidelines | 1. Yes  2. No  3. Don’t Know | Miongozo ya udhibiti na matibabu ya magonjwa ya zinaa | | 1. Ndiyo  2. Hapana  3. Sifahamu |
| Confidentiality guidelines | 1. Yes  2. No  3. Don’t Know | Muongozo wa usiri | | 1. Ndiyo  2. Hapana  3. Sifahamu |
| Informed consent guidelines | 1. Yes  2. No  3. Don’t Know | Muongozo wa utoaji ridhaa | | 1. Ndiyo  2. Hapana  3. Sifahamu |
| Post exposure prophylaxis guidelines | 1. Yes  2. No  3. Don’t Know | Muongozo wa dawa za kupunguza uwezekano wa kupata VVU baada ya kugusana na VVU (post-exposure prophylaxis au PEP guidelines) | | 1. Ndiyo  2. Hapana  3. Sifahamu |
| Guidelines related to working with adolescents | 1. Yes  2. No  3. Don’t Know | Miongozo inayohusiana na kuhudumia vijana | | 1. Ndiyo  2. Hapana  3. Sifahamu |
| ***Provider Training Adequacy Scale items*** | | | | |
| Providers have been sufficiently trained: |  | Watoa huduma wamepata mafunzo ya kutosha katika; | |  |
| to provide HIV prevention and treatment to women | 1. None 2. Some 3. Most 4. All | Kutoa huduma za kinga na matibabu ya VVU kwa wanawake | | 1. Hakuna 2. Baadhi 3. Wengi 4. Wote |
| to counsel patients on HIV prevention | 1. None 2. Some 3. Most 4. All | Kushauri wagonjwa kuhusu kujikinga na VVU | | 1. Hakuna 2. Baadhi 3. Wengi 4. Wote |
| to counsel patients on adherence. | 1. None 2. Some 3. Most 4. All | Kushauri wagonjwa kuhusu kuzingatia matumizi sahihi ya dawa | | 1. Hakuna 2. Baadhi 3. Wengi 4. Wote |
| to respond to the needs of adolescent girls and young women. | 1. None 2. Some 3. Most 4. All | Kuzingatia mahitaji ya vijana wa kike na wanawake wenye umri mdogo. | | 1. Hakuna 2. Baadhi 3. Wengi 4. Wote |
| to provide HIV treatment to the adolescent and young adult population | 1. None 2. Some 3. Most 4. All | Kutoa tiba ya VVU kwa vijana na watu wazima wenye umri mdogo. | | 1. Hakuna 2. Baadhi 3. Wengi 4. Wote |
| to provide HIV prevention services to the adolescent and young adult population | 1. None 2. Some 3. Most 4. All | Kutoa huduma za kuzuia maambukizi ya VVU kwa vijana na watu wazima wenye umri mdogo. | | 1. Hakuna 2. Baadhi 3. Wengi 4. Wote |
| **Accessibility** |  |  | |  |
| Are there services available at this facility that specifically target adolescents and young adults? | 1. Yes  2. No  88. Don’t Know | Je, kuna huduma zinazopatikana kituoni hapa ambazo zinalenga hasa vijana na watu wazima wenye umri mdogo? | | 1. Ndiyo  2. Hapana  88. Sifahamu |
| **Efficient and effectively organized care** |  |  | |  |
| On average, how long does a client wait before being seen by a provider (e.g. counselor, nurse, doctor)? Please give a best guess. | 1. Less than 15 minutes 2. Between 15-30 minutes 3. Between 31-60 minutes 4. More than 60 minutes | Je, kwa wastani, ni muda gani mteja husubiri kabla ya kuonana na mtoa huduma (mfano mshauri nasaha, muuguzi, ddaktari)? Tafadhali nipe makadirio yako bora zaidi. | | 1. Chini ya dakika 15 2. Kati ya dakika 15-30 3. Kati ya dakika 31-60   4. Zaidi ya dakika 60 |
| Are there protocols in place to follow-up clients who need additional care? | 1. Yes  2. No  88. Don’t Know | Je, kuna itifaki zilizopo za kufuatilia wateja wanaohitaji huduma za ziada? | | 1. Ndiyo  2. Hapana  88. Sifahamu |
| ***PrEP Service Impact Scale items*** |  |  | |  |
| I do not have sufficient time to provide HIV prevention counseling to my current patients so providing PrEP should wait a few years. | 1. Strongly disagree  2. Disagree  3. Neither agree nor disagree  4. Agree  5. Strongly agree | Sina muda wa kutosha kutoa ushauri nasaha wa kujikinga na VVU kwa wagonjwa nilionao hivi sasa, kwa hiyo utoaji wa huduma ya PrEP inabidi usubiri kwa miaka kadhaa. | | 1. Sikubaliani kabisa 2. Sikubaliani 3. Siko upande wowote  4. Nakubaliana 5. Nakubaliana Kabisa |
| I do not have time to provide clinical monitoring required with providing PrEP | 1. Strongly disagree  2. Disagree  3. Neither agree nor disagree  4. Agree  5. Strongly agree | Sina muda wa kutosha wa kufanya ufuatiliaji wa kitabibu unaotakiwa katika utoaji wa PrEP | | 1. Sikubaliani kabisa 2. Sikubaliani 3. Siko upande wowote  4. Nakubaliana 5. Nakubaliana Kabisa |
| The use of PrEP for prevention will result in less availability of HIV drugs for people living with HIV | 1. Strongly disagree  2. Disagree  3. Neither agree nor disagree  4. Agree  5. Strongly agree | Matumizi ya PrEP kwa ajili ya kinga husababisha kupungua kwa upatikanaji wa dawa za VVU kwa watu waishio na VVU | | 1. Sikubaliani kabisa 2. Sikubaliani 3. Siko upande wowote  4. Nakubaliana 5. Nakubaliana Kabisa |
| I am uncomfortable prescribing PrEP unless we know more about long term side effects. | 1. Strongly disagree  2. Disagree  3. Neither agree nor disagree  4. Agree  5. Strongly agree | Sijisikii vizuri kutoa PrEP hadi pale ambapo tutafahamu zaidi kuhusu madhara ya muda mrefu. | | 1. Sikubaliani kabisa 2. Sikubaliani 3. Siko upande wowote  4. Nakubaliana 5. Nakubaliana Kabisa |
| I am uncomfortable providing PrEP until we know more about the drug’s effectiveness to prevent HIV. | 1. Strongly disagree  2. Disagree  3. Neither agree nor disagree  4. Agree  5. Strongly agree | Sijisikii vizuri kutoa PrEP hadi pale ambapo tutafahamu zaidi kuhusu ufanisi wa dawa hii kwenye kuzuia VVU. | | 1. Sikubaliani kabisa 2. Sikubaliani 3. Siko upande wowote  4. Nakubaliana 5. Nakubaliana Kabisa |
| **Structure and facilities** |  |  | |  |
| The waiting rooms are often crowded at this facility. | 1. Strongly disagree 2. Disagree 3. Agree 4. Strongly agree | Mara nyingi, vyumba vya kusubiria vina watu wengi kwenye kituo hiki. | | 1. Sikubaliani kabisa 2. Sikubaliani 3. Nakubaliana 4. Nakubaliana Kabisa |
| **Appropriate package of services** |  |  | |  |
| This facility has experienced stock outs of the following HIV prevention/treatment options in the last 12 months. |  | Kituo hiki kimewahi kuishiwa kabisa nyenzo zifuatazo za kuzuia/kutibu VVU ndani ya miezi 12 iliyopita. | |  |
| Condoms | 1. Yes 2. No 3. Don’t Know   99. Not applicable | Kondomu | | 1. Ndiyo  2. Hapana  88. Sifahamu  99. Haihusiki |
| HIV rapid tests | 1. Yes  2. No  88. Don’t Know  99. Not applicable | Vipimo vya haraka vya VVU | | 1. Ndiyo  2. Hapana  88. Sifahamu  99. Haihusiki |
| HIV confirmatory tests | 1. Yes  2. No  88. Don’t Know  99. Not applicable | Vipimo vya kuhakikisha maambukizi ya VVU | | 1. Ndiyo  2. Hapana  88. Sifahamu  99. Haikusiki |
| STI tests | 1. Yes  2. No  88. Don’t Know  99. Not applicable | Vipimo vya magonjwa ya zinaa | | 1. Ndiyo  2. Hapana  88. Sifahamu  99. Haihisuki |
| Anti-retroviral therapy drugs | 1. Yes  2. No  88. Don’t Know  99. Not applicable | Dawa za kupunguza makali ya VVU | | 1. Ndiyo  2. Hapana  88. Sifahamu  99. Haihusiki |
| Test kits for CD4 and viral load | 1. Yes  2. No  88. Don’t Know  99. Not applicable | Vipimo vya CD4 na wingi wa virusi | | 1. Ndiyo  2. Hapana  88. Sifahamu  99. Haihusiki |
| This facility has a system to prevent stock outs of supplies | 1. Strongly disagree 2. Disagree 3. Agree 4. Strongly agree | Kituo hiki kina mfumo wa kuzuia vifaa kuisha kabisa | | 1. Sikubaliani kabisa 2. Sikubaliani 3. Nakubaliana 4. Nakubaliana Kabisa |
| *Additional question used in paper* |  |  | |  |
| Providers have sufficient time to talk to each client about their medical history | 1. Strongly disagree 2. Disagree 3. Agree 4. Strongly agree | Watoa huduma wana muda wa kutosha kuongea na kila mteja kuhusu historia yao ya matibabu | | 1. Sikubaliani kabisa 2. Sikubaliani 3. Nakubaliana 4. Nakubaliana Kabisa |
| **Outcome** |  |  | |  |
| If PrEP became available at this facility today, which of the following sub-populations of adolescent girls and young women would you provide PrEP if you come in contact with them [record response for each] | 1. Adolescent girls and young women with a sexually transmitted disease 2. Adolescent girls and young women who change sex partners frequently 3. Adolescent girls and young women who exchange sex for money, drugs or other goods or services (e.g., school fees, employment, transportation) 4. Adolescent girls and young women with a history of abuse from their partner 5. Adolescent girls and young women with HIV-positive partners | Kama PrEP ingepatikana kwenye kituo hiki leo, ni makundi gani ya vijana wa kike na wanawake wenye umri mdogo ambayo ungewapatia dawa hii kama ungekutana nao [weka alama kwa zote zinazohusika] | | 1. Vijana wa kike na wanawake wenye umri mdogo wenye gonjwa la zinaa 2. Vijana wa kike na wanawake wenye umri mdogo wanaobadilisha wenzi wao mara kwa mara 3. Vijana wa kike na wanawake wenye umri mdogo wanaouza miili yao kwa ajili ya kupata fedha, madawa ya kulevya, au bidhaa zingine (mfano ada ya shule, ajira, usafiri) 4. Vijana wa kike na wanawake wenye umri mdogo wenye historia ya kufanyiwa unyanyasaji na wenzi wao. 5. Vijana wa kike na wanawake wenye umri mdogo ambao wana wenzi wenye maambukizi ya VVU. |
| **Demographic Characteristics** |  |  | |  |
| Type of facility | 1. Hospital 2. Health Center 3. Dispensary   Clinic | Aina ya kituo cha afya | | 1. Hospitali 2. Kituo cha afya 3. Zahanati   Kliniki |
| Facility managing authority | 1. Private 2. Public 3. Faith-based organization 4. Military   Parastatal facilities | Uongozi wa Kituo | | 1. Binafsi 2. Umma 3. Shirika la Kidini 4. Jeshi   Vituo vya Mashirika ya Umma |
| What is your age in years? | _______ | Je, una umri gani (katika miaka iliyo kamilika)? | | _______ |
| What is your sex? | 1. Male   Female | Je, jinsia yako ni ipi? | | 1. Kiume   Kike |
| What is your main profession at this facility? | 1. Nurse 2. Doctor 3. Clinical officer 4. Counselor   Other (specify) | Je, kazi yako kuu ni ipi kwenye kituo hiki? | | 1. Muuguzi 2. Daktari 3. Daktari msaidizi 4. Mshauri Nasaha   Nyingine (bainisha) |
| How many years have you worked in this profession? Please answer in complete years. | _________ years  99. Less than one year | Je, umefanya kazi kwa miaka mingapi kwenye taaluma hii? [Tafadhali jibu katika miaka iliyo kamilika] | | Miaka_________  99. Chini ya mwaka mmoja |
| How many people living with HIV do you attend to in a typical week? Please give your best estimate. | _______ people | Je, kwa kawaidia ni watu wangapi waishio na VVU unaowahudumia ndani ya wiki moja?  [Tafadhali toa makadirio unayohisi ni bora Zaidi] | | Watu_______ |
| One of the medicines in oral PrEP is tenofovir. | 1. True 2. False   88. Don’t know | Moja kati ya dawa zilizopo kwenye PrEP inayotumiwa kwa njia ya kinywa ni tenofovir. | | 1. Kweli 2. Si kweli  88. Sifahamu |
| It is currently recommended that oral PrEP be taken once a day at the same time each day. | 1. True 2. False   88. Don’t know | Kwa sasa inapendekezwa PrEP imezwe mara moja kwa siku muda ule ule kila siku. | | 1. Kweli 2. Si kweli  88. Sifahamu |
| HIV positive people can take oral PrEP. | 1. True 2. False   88. Don’t know | Watu wenye maambukizi ya VVU wanaweza kunywa PrEP. | | 1. Kweli 2. Si kweli  88. Sifahamu |
| Both men and women can use oral PrEP. | 1. True 2. False   88. Don’t know | Wanawake na wanaume wanaweza kutumia PrEP | | 1. Kweli 2. Si kweli  88. Sifahamu |
| Oral PrEP does not protect against pregnancy | 1. True 2. False   88. Don’t know | Kunywa PrEP hakuzuii mtu kupata ujauzito | | 1. Kweli 2. Si kweli  88. Sifahamu |
| Oral PrEP protects against other sexually transmitted infections (STI) besides HIV | 1. True 2. False   88. Don’t know | Mbali na ulinzi dhidi ya VVU, unywaji wa PrEP hutoa ulinzi dhidi ya magonjwa mengine ya zinaa | | 1. Kweli 2. Si kweli  88. Sifahamu |
| People who engage in behaviors that put them at high risk of HIV should not take oral PrEP. | 1. True 2. False   88. Don’t know | Watu wanaojihusisha na tabia zinazowaweka kwenye hatari kubwa ya kupata VVU hawapaswi kunywa PrEP. | | 1. Kweli 2. Si kweli  88. Sifahamu |
| Viral resistance to PrEP drug(s) can arise if the person starts PrEP with undiagnosed HIV. | 1. True 2. False   88. Don’t know | Mwili unaweza kujenga usugu kwa dawa ya PrEP kama mtu ataanza kuinywa dawa hiyo akiwa na maambukizi ya VVU ambayo bado hayajagunduliwa. | | 1. Kweli 2. Si kweli  88. Sifahamu |
| An HIV-negative person can take oral PrEP even if their HIV-positive partner is taking HIV medication. | 1. True 2. False   88. Don’t know | Mtu ambaye hana maambukizi ya VVU anaweza kunywa PrEP hata kama mwenzi wake mwenye VVU anatumia dawa za VVU. | | 1. Kweli 2. Si kweli  88. Sifahamu |
| ^1^ In the survey, these items were designed to capture the domain of client-staff interaction. They loaded with the Patient-Centeredness Scale.  ^2^ In the survey, these items were designed to capture the domain of communication and information. They loaded with the Patient-Centeredness Scale. | | | | |
