# Supplementary material for: Provider perspectives on PrEP for adolescent girls and young women in Tanzania: The role of provider biases and quality of care
Source: PLoS One. 2018 Apr 27;13(4):e0196280. doi: 10.1371/journal.pone.0196280 (PMC5922529; doi:10.1371/journal.pone.0196280)
Supplement: S2 Table — (DOCX) [file pone.0196280.s002.docx]

**Supporting Information 2**

| **Qualitative Questions, Codes and Definitions Used to Assess Domains of Quality of Care** | | |
| --- | --- | --- |
| **Domain** | **Questions** | **Codes and Definitions*** |
| **Provider-level** | |  |
| **Patient-centered care** | In general, how would you describe providers’ attitudes and perceptions towards providing sexual and reproductive health services to adolescent and young adults? [Probe: How do these attitudes and perceptions differ based on: gender, marital status age]  What are your opinions about making HIV PrEP available to adolescent girls and young women? [Probe based on age, marital status, sexual behavior] | **HealthStaffInteract:** Comments on how the service provider positive or negative interactions with their patients. This also includes perceptions of the interviewed provider of their colleagues engagement with patients.  **BehaviouralDisin:** Any statements referring to how PrEP use will increase, decrease or not change high risk behavior and reasons why.  **HealthsystemUtil:** Service providers’ descriptions of how the health system is being utilized or not utilized and what impacts health service utilization.  **HealthSystemUtil-Culture:**  Statements by service providers of how cultural and gender norms affect the utilization of or provision of health services. |
| **Client-staff interactions** | Would you feel comfortable prescribing PrEP? Why or why not?  What about to adolescent girls and young women? | **HealthStaffPrEP:** Any comments made by service providers on how they will impact, whether positively or negatively, the uptake of PrEP by different patients.  **HealthStaffInteract:** Comments on how the service provider positive or negative interactions with their patients. This also includes perceptions of the interviewed provider of their colleagues engagement with patients. |
| **Communication and Information** | How can you support women’s use of PrEP or other daily ARV-based medication?  In what ways would you help women understand and manage potential side effects of PrEP? | **PrEPAdvocacy:** Statements by providers on how they will advocate for the use of PrEP to patients.  **HealthStaffPrEP:** Any comments or statements referring to how any actor involved with the health system would affect use and adherence to PrEP. This includes influencing decision to use PrEP, support continued adherence to PrEP, advertise or prescribe or inform about PrEP. |
| **Technically competent care** | How well do you think this clinic/health facility is currently doing in providing HIV and other sexual/reproductive health services for adolescents?  How can service delivery be improved?  How do you/your clinic, currently measure/assess if clients are adhering to their ARV medications? | **HealthSystemChall-Staff:** Statements by service providers outlining any challenge faced by any health staff in the health system  **HealthStaffPrEP-Infra:** Statements by the service providers on what kinds of health personnel, equipment, materials and other infrastructural support will be needed to make PrEP available and to ensure its uptake. |
| **Facility-level** | |  |
| **Accessibility** | What do you consider appropriate service-delivery settings to offer PrEP to adolescent girls and young women? Why? | **Location-NotPrefPrEp: P**laces where providers did not think PrEP should be provided or where they think clients would not want to access to PrEP and reasons why.  **Location-PrefPrEp: P**laces where providers think PrEP should be provided or where they think clients would want to access to PrEP and reasons why. |
| **Efficient and effectively organized care** | In your opinion, what cadre(s) of providers is needed to ensure appropriate counseling, provision, testing, resupply, and follow-up if PrEP was introduced for adolescent girls and young women? Why?  What kind of support will health staff need at this facility to provide PrEP?  At your clinic/health facility, how would providing PrEP affect exist­ing services? | **HealthStaffPrEP-Infra:** Statements by the service providers on what kinds of health personnel, equipment, materials and other infrastructural support will be needed to make PrEP available and to ensure its uptake.  **HealthSystemChall-Infra:** Any comments about structural issues that provide a challenge to providers providing optimal care to clients.  **ImpactPrEP-HealthSystems:** Any comments made by the service provider on how the roll out of or making available PrEP will impact the health system positively (including not having any effect) or negatively. |
| **Structure and facilities** | Where do you think adolescent girls and young women would not feel comfortable accessing PrEP services? Why? | **Location-NotPrefPrEp:** Places where providers did not think PrEP should be provided or where they think clients would not want to access to PrEP and reasons why.  **Location-PrefPrEp:** Places where providers think PrEP should be provided or where they think clients would want to access to PrEP and reasons why. |
| **Appropriate package of services** | What kind of sexual and reproductive health services do you provide to adolescents and young adult? | **HealthServices-SRH:** Any kind of sexual and reproductive health services that are provided or not provided as well as services that the provider desires to provide.  **NeedforPrEP:** What circumstances create a need for PrEP amongst their patient populations. |
| * Some codes apply to multiple domains because providers discussed several domains together. | | |
