# Supplementary material for: Provider perspectives on PrEP for adolescent girls and young women in Tanzania: The role of provider biases and quality of care
Source: PLoS One. 2018 Apr 27;13(4):e0196280. doi: 10.1371/journal.pone.0196280 (PMC5922529; doi:10.1371/journal.pone.0196280)
Supplement: S3 Table — (DOCX) [file pone.0196280.s003.docx]

**SUPPLEMENTAL INFORMATION 3 - Quality of Care Measures by Provider Sex**

|  | **Male**  **(n=91)**  **% or mean (sd)** | **Female**  **(n=225)**  **% or mean (sd)** | **p-value** |
| --- | --- | --- | --- |
| **Provider-level** | | | |
| **Patient-Centered Care** |  |  |  |
| Negative Attitudes towards Adolescent Sexuality | 18.3 (4.8) | 19.8 (5.9) | 0.007 |
| Behavioral Disinhibition Scale | 10.6 (3.8) | 11.2 (4.1) | 0.198 |
| Patient-Centered Scale | 34.6 (4.0) | 34.9 (3.8) | 0.443 |
| **Technically Competent Care** |  |  |  |
| Provider Training Adequacy Scale | 13.8 (3.4) | 14.4 (3.8) | 0.117 |
| Has access to HIV guidelines |  |  | 0.072 |
| No | 41.8 | 32.4 |  |
| Yes | 58.2 | 67.5 |  |
| **Facility-level** | | | |
| **Accessibility** |  |  |  |
| Facility has services focused on adolescents and young adults |  |  | 0.018 |
| No/don't know | 28.6 | 16.4 |  |
| Yes | 71.4 | 83.6 |  |
| **Efficient and effectively organized care** |  |  |  |
| PrEP Service Impact Scale | 11.3 (3.9) | 11.7 (4.1) | 0.007 |
| Client waiting time at facility |  |  | 0.692 |
| Less than 15 minutes | 38.5 | 39.6 |  |
| Between 15-30 minutes | 53.9 | 49.8 |  |
| Greater than 30 minutes | 7.7 | 10.7 |  |
| Protocols in place for client follow-up |  |  | 0.231 |
| No | 15.4 | 21.3 |  |
| Yes | 84.6 | 78.7 |  |
| **Structure and facilities** |  |  |  |
| Crowded waiting rooms |  |  | 0.485 |
| Disagree | 49.5 | 53.8 |  |
| Agree | 50.5 | 46.2 |  |
| **Appropriate package of services** |  |  |  |
| Facility had stock-outs of HIV prevention and treatment options in last 12 months |  |  | 0.023 |
| No | 49.5 | 62.2 |  |
| Yes | 50.5 | 37.8 |  |
| Facility has system to prevent stockouts of supplies |  |  | 0.733 |
| Disagree | 23.1 | 24.9 |  |
| Agree | 76.9 | 75.1 |  |
