# Supplementary material for: Provider perspectives on PrEP for adolescent girls and young women in Tanzania: The role of provider biases and quality of care
Source: PLoS One. 2018 Apr 27;13(4):e0196280. doi: 10.1371/journal.pone.0196280 (PMC5922529; doi:10.1371/journal.pone.0196280)
Supplement: S4 Table — (DOCX) [file pone.0196280.s004.docx]

**SUPPLEMENTAL INFORMATION 4 – Quality of Care Measures by Provider Profession**

|  | **Nurse**  **(n=154)**  **% or mean (sd)** | **Doctor**  **(n=48)**  **% or mean (sd)** | **Clinical Officer**  **(n=39)**  **% or mean (sd)** | **Counselor**  **(n=41)**  **% or mean (sd)** | **Other**  **(N=34)**  **% or mean (sd)** | **p-value** |
| --- | --- | --- | --- | --- | --- | --- |
| **Provider-Level** | | | | | | |
| **Patient-Centered Care** |  |  |  |  |  |  |
| Negative Attitudes towards Adolescent Sexuality | 19.5 (6.0) | 18.6 (5.2) | 17.9 (4.4) | 20.2 (5.8) | 21.0 (5.3) | ns |
| Behavioral Disinhibition Scale | 11.2 (4.2) | 11.0 (4.1) | 10.4 (3.4) | 10.6 (3.3) | 11.2 (4.4) | ns |
| Patient-Centered Scale | 35.2 (3.8) | 33.9 (3.8) | 35.6 (3.9) | 34.6 (3.9) | 34.0 (3.6) | ns |
| **Technically Competent Care** |  |  |  |  |  |  |
| Provider Training Adequacy Scale | 14.5 (3.6) | 13.3 (3.0) | 14.4 (3.9) | 13.9 (3.6) | 14.6 (4.2) | <0.05^a^ |
| Has access to HIV guidelines |  |  |  |  |  | 0.310 |
| No | 30.5 | 37.5 | 48.7 | 36.6 | 35.3 |  |
| Yes | 69.5 | 62.5 | 51.2 | 63.4 | 64.7 |  |
| **Facility-Level** | | | | | | |
| **Accessibility** |  |  |  |  |  |  |
| Facility has services focused on adolescents and young adults |  |  |  |  |  | 0.101 |
| No/don't know | 14.9 | 25.0 | 33.3 | 17.1 | 23.5 |  |
| Yes | 85.1 | 75.0 | 66.7 | 82.9 | 76.5 |  |
| **Efficient and effectively organized care** |  |  |  |  |  |  |
| PrEP Service Impact Scale | 11.5 (4.1) | 11.2 (3.8) | 9.6 (3.9) | 11.7 (3.8) | 11.9 (4.3) | <0.01^b^ |
| Client waiting time at facility |  |  |  |  |  | 0.263 |
| Less than 15 minutes | 43.5 | 35.4 | 35.9 | 46.3 | 20.6 |  |
| Between 15-30 minutes | 45.5 | 54.3 | 53.9 | 46.3 | 73.5 |  |
| Greater than 30 minutes | 11.0 | 10.4 | 10.3 | 7.3 | 5.9 |  |
| Protocols in place for client follow-up |  |  |  |  |  | 0.606 |
| No | 22.7 | 14.6 | 18.0 | 22.0 | 11.8 |  |
| Yes | 77.3 | 85.4 | 82.0 | 78.0 | 88.2 |  |
| **Structure and facilities** |  |  |  |  |  |  |
| Crowded waiting rooms |  |  |  |  |  | 0.317 |
| Disagree | 50.0 | 52.1 | 48.7 | 51.2 | 70.6 |  |
| Agree | 50.0 | 47.9 | 51.3 | 48.8 | 29.4 |  |
| **Appropriate package of services** |  |  |  |  |  |  |
| Facility had stock-outs of HIV prevention and treatment options in last 12 months |  |  |  |  |  | 0.053 |
| No | 66.2 | 50.0 | 48.7 | 61.0 | 44.1 |  |
| Yes | 33.8 | 50.0 | 51.3 | 39.0 | 55.9 |  |
| Facility has system to prevent stockouts of supplies |  |  |  |  |  | 0.368 |
| Disagree | 24.0 | 27.1 | 17.9 | 35.3 | 24.4 |  |
| Agree | 76.0 | 72.9 | 82.1 | 64.7 | 75.6 |  |
| *NS indicate that the mean of the scales did not significantly differently differ across the provider type  ^a^ Significant when comparing doctors to nurses  ^b^ Significant when comparing clinical officers to nurses | | | | | | |
