# Supplementary material for: Provider perspectives on PrEP for adolescent girls and young women in Tanzania: The role of provider biases and quality of care
Source: PLoS One. 2018 Apr 27;13(4):e0196280. doi: 10.1371/journal.pone.0196280 (PMC5922529; doi:10.1371/journal.pone.0196280)
Supplement: S5 Table — (DOCX) [file pone.0196280.s005.docx]

**SUPPLEMENTAL INFORMATION 5 - Quality of Care Measures by Region**

|  | **Dar Es Salaam**  **(n=196)**  **% or mean (sd)** | **Mbeya**  **(n=120)**  **% or mean (sd)** | **p-value** |
| --- | --- | --- | --- |
| **Provider-Level** | | | |
| **Patient-Centered Care** |  |  |  |
| Negative Attitudes towards Adolescent Sexuality | 20.3 (6.0) | 18.4 (4.9) | 0.036 |
| Behavioral Disinhibition Scale | 10.9 (4.1) | 11.0 (3.9) | 0.868 |
| Patient-Centered Scale | 34.5 (3.9) | 35.4 (3.8) | 0.097 |
| **Technically Competent Care** |  |  |  |
| Provider Training Adequacy Scale | 14.2 (3.3) | 14.3 (4.2) | 0.868 |
| Has access to HIV guidelines |  |  | 0.439 |
| No | 33.2 | 38.3 |  |
| Yes | 66.7 | 61.7 |  |
| **Facility-Level** | | | |
| **Accessibility** |  |  |  |
| Facility has services focused on adolescents and young adults |  |  | 0.806 |
| No/don't know | 20.4 | 19.2 |  |
| Yes | 79.6 | 80.8 |  |
| **Efficient and effectively organized care** |  |  |  |
| PrEP Service Impact Scale | 12.4 (3.8) | 10.1 (4.1) | <0.001 |
| Client waiting time at facility |  |  | 0.128 |
| Less than 15 minutes | 43.9 | 31.7 |  |
| Between 15-30 minutes | 46.9 | 57.5 |  |
| Greater than 30 minutes | 9.2 | 10.8 |  |
| Protocols in place for client follow-up |  |  | 0.642 |
| No | 20.4 | 18.3 |  |
| Yes | 79.6 | 81.7 |  |
| **Structure and facilities** |  |  |  |
| Crowded waiting rooms |  |  | 0.574 |
| Disagree | 51.0 | 55.0 |  |
| Agree | 49.0 | 45.0 |  |
| **Appropriate package of services** |  |  |  |
| Facility had stock-outs of HIV prevention and treatment options in last 12 months |  |  | 0.005 |
| No | 66.3 | 45.8 |  |
| Yes | 33.7 | 54.2 |  |
| Facility has system to prevent stockouts of supplies |  |  | 0.853 |
| Disagree | 23.9 | 25.0 |  |
| Agree | 76.0 | 75.0 |  |
