# Supplementary material for: Provider perspectives on PrEP for adolescent girls and young women in Tanzania: The role of provider biases and quality of care
Source: PLoS One. 2018 Apr 27;13(4):e0196280. doi: 10.1371/journal.pone.0196280 (PMC5922529; doi:10.1371/journal.pone.0196280)
Supplement: S6 Table — (DOCX) [file pone.0196280.s006.docx]

**SUPPLEMENTAL INFORMATION 6 – Quality of Care Measures by Facility Type**

|  | **Hospital**  **(n=66)**  **% or mean (sd)** | **Health Center**  **(n=66)**  **% or mean (sd)** | **Dispensary**  **(n=184)**  **% or mean (sd)** | **p-value** |
| --- | --- | --- | --- | --- |
| **Provider-Level** | | | | |
| **Patient-Centered Care** |  |  |  |  |
| Negative Attitudes towards Adolescent Sexuality | 20.8 (6.7) | 18.8 (5.4) | 19.1 (5.3) | ns* |
| Behavioral Disinhibition Scale | 12.0 (4.0) | 10.1 (4.1) | 11.0 (3.9) | 0.014^a^ |
| Patient-Centered Scale | 34.9 (4.1) | 34.4 (3.8) | 34.9 (3.8) | ns |
| **Technically Competent Care** |  |  |  |  |
| Provider Training Adequacy Scale | 14.9 (3.3) | 13.6 (3.1) | 14.2 (3.9) | 0.017^a^ |
| Has access to HIV guidelines |  |  |  | 0.104 |
| No | 27.3 | 27.3 | 40.8 |  |
| Yes | 72.7 | 72.7 | 59.2 |  |
| **Facility-Level** | | | | |
| **Accessibility** |  |  |  |  |
| Facility has services focused on adolescents and young adults |  |  |  | 0.303 |
| No/don't know | 21.2 | 12.1 | 22.3 |  |
| Yes | 78.8 | 87.9 | 11.7 |  |
| **Efficient and effectively organized care** |  |  |  |  |
| PrEP Service Impact Scale | 11.9 (3.8) | 11.1 (4.2) | 11.2 (4.6) | ns |
| Client waiting time at facility |  |  |  | 0.589 |
| Less than 15 minutes | 33.3 | 39.4 | 41.3 |  |
| Between 15-30 minutes | 53.0 | 50.0 | 50.5 |  |
| Greater than 30 minutes | 13.6 | 10.6 | 8.2 |  |
| Protocols in place for client follow-up |  |  |  | 0.180 |
| No | 27.3 | 13.6 | 19.0 |  |
| Yes | 72.7 | 86.4 | 80.0 |  |
| **Structure and facility** |  |  |  |  |
| Crowded waiting rooms |  |  |  | 0.777 |
| Disagree | 50.0 | 50.0 | 54.4 |  |
| Agree | 50.0 | 50.0 | 45.6 |  |
| **Appropriate package of services** |  |  |  |  |
| Facility had stock-outs of HIV prevention and treatment options in last 12 months |  |  |  | 0.314 |
| No | 63.6 | 63.6 | 54.9 |  |
| Yes | 36.4 | 36.4 | 45.1 |  |
| Facility has system to prevent stockouts of supplies |  |  |  | 61.7 |
| Disagree | 19.7 | 24.2 | 26.1 |  |
| Agree | 80.3 | 75.8 | 73.9 |  |
| *NS indicate that the mean of the scales did not significantly differently differ across the three facility types  ^a^ Significant when comparing health centers to hospitals | | | |  |
