# Supplementary material for: Provider perspectives on PrEP for adolescent girls and young women in Tanzania: The role of provider biases and quality of care
Source: PLoS One. 2018 Apr 27;13(4):e0196280. doi: 10.1371/journal.pone.0196280 (PMC5922529; doi:10.1371/journal.pone.0196280)
Supplement: S7 Table — (DOCX) [file pone.0196280.s007.docx]

**SUPPLEMENTAL INFORMATION 7 – Quality of Care Measures by Facility Managing Authority**

|  | **Private**  **(n=56)**  **% or mean (sd)** | **Public**  **(n=192)**  **% or mean (sd)** | **FBO**  **(n=42)**  **% or mean (sd)** | **Military**  **(n=11)**  **% or mean (sd)** | **Parastalal (N=15)**  **% or mean (sd)** | **p-value** |
| --- | --- | --- | --- | --- | --- | --- |
| **Provider-Level** | | | | | | |
| **Patient-Centered Care** |  |  |  |  |  |  |
| Negative Attitudes towards Adolescent Sexuality | 21.1 (5.8) | 18.8 (5.0) | 19.6 (5.9) | 25.4 (9.4) | 16.8 (4.3) | p<0.05^a^ |
| Behavioral Disinhibition Scale | 10.9 (4.0) | 11.0 (3.9) | 10.4 (4.0) | 13.4 (4.6) | 10.5 (4.0) | p<0.001^b^ |
| Patient-Centered Scale | 34.1 (3.8) | 34.7 (3.9) | 35.8 (3.7) | 36.7 (3.4) | 34.8 (3.9) | p<0.001^c^ |
| **Technically Competent Care** |  |  |  |  |  |  |
| Provider Training Adequacy Scale | 13.9 (3.7) | 14.3 (3.8) | 13.6 (2.7) | 14.2 (2.3) | 15.4 (4.8) | ns |
| Has access to HIV guidelines |  |  |  |  |  | 0.407 |
| No | 37.5 | 31.8 | 47.6 | 36.4 | 33.3 |  |
| Yes | 62.5 | 68.2 | 52.4 | 63.6 | 67.7 |  |
| **Facility-Level** | | | | | | |
| **Accessibility** |  |  |  |  |  |  |
| Facility has services focused on adolescents and young adults |  |  |  |  |  | 0.521 |
| No/don't know | 21.4 | 17.7 | 21.4 | 36.4 | 26.7 |  |
| Yes | 78.6 | 82.3 | 78.6 | 63.6 | 73.3 |  |
| **Efficient and effectively organized care** |  |  |  |  |  |  |
| PrEP Service Impact Scale | 12.2 (3.7) | 11.1 (3.9) | 10.4 (4.2) | 13.7 (3.0) | 10.8 (4.7) | p<0.05^c^ |
| Client waiting time at facility |  |  |  |  |  | 0.197 |
| Less than 15 minutes | 48.2 | 36.5 | 45.2 | 27.3 | 33.3 |  |
| Between 15-30 minutes | 46.4 | 51.0 | 52.4 | 64.6 | 53.3 |  |
| Greater than 30 minutes | 5.4 | 12.5 | 2.4 | 9.1 | 13.3 |  |
| Protocols in place for client follow-up |  |  |  |  |  | 0.189 |
| No | 17.9 | 20.3 | 14.3 | 45.5 | 13.3 |  |
| Yes | 82.1 | 79.7 | 85.7 | 54.6 | 86.7 |  |
| **Structure and facilities** |  |  |  |  |  |  |
| Crowded waiting rooms |  |  |  |  |  | 0.098 |
| Disagree | 64.3 | 46.9 | 54.8 | 54.6 | 73.3 |  |
| Agree | 35.7 | 53.1 | 45.2 | 45.5 | 26.7 |  |
| **Appropriate package of services** |  |  |  |  |  |  |
| Facility had stock-outs of HIV prevention and treatment options in last 12 months |  |  |  |  |  | 0.359 |
| No | 62.5 | 58.9 | 61.9 | 27.3 | 53.3 |  |
| Yes | 37.5 | 41.2 | 38.1 | 72.7 | 56.7 |  |
| Facility has system to prevent stockouts of supplies |  |  |  |  |  | 0.444 |
| Disagree | 25.0 | 27.6 | 19.1 | 0.0 | 13.3 |  |
| Agree | 75.0 | 72.0 | 81.0 | 100.0 | 86.7 |  |
| *NS indicate that the mean of the scales did not significantly differently differ across the managing authority of facility  ^a^ Significant when comparing military and parastalal facilities to private facilities  ^b^ Significant when comparing military to private facilities  ^c^ Significant when comparing military and FBO facilities to private facilities | | | | | | |
